# Relevance of the regional lymph node in scrapie pathogenesis after peripheral infection of hamsters

**DOI:** 10.1186/1746-6148-3-22

**Published:** 2007-09-25

**Authors:** Christine Kratzel, Dominique Krüger, Michael Beekes

**Affiliations:** 1 Robert Koch-Institut, P24 – Transmissible Spongiforme Enzephalopathien Nordufer 20, D-Berlin 13353, Germany

## Abstract

**Background:**

The exact role of the lymphoreticular system in the spread of peripheral prion infections to the central nervous system still needs further elucidation. Against this background, the influence of the regional lymph node *(Ln. popliteus*) on the pathogenesis of scrapie was monitored in a hamster model of prion infection *via *the footpad.

**Methods:**

Surgical lymphadenectomy was carried out at different time points after infection, or prior to inoculation, in order to elucidate the impact of the lymph node on lethal neuroinvasion.

**Results:**

The *Ln. popliteus *did not show an influence on pathogenesis when a high dose of infectivity was administered. However, it was found to modulate the interval of time until the development of terminal scrapie in a subset of animals lymphadenectomized after low-dose infection. In additon, lymphadenectomy performed four weeks before inoculation prevented cerebral PrP^TSE ^deposition and development of disease during the period of observation (314 days) in the majority of hamsters challenged with a very low dose of scrapie agent.

**Conclusion:**

Our findings suggest the regional lymph node as a potentially facilitating or even essential factor for invasion of the brain after peripheral challenge with low doses of infectious scrapie agent. The invasive *in vivo *approach pursued in this study may be applied also to other animal species for further elucidating the involvement of lymphoid tissue in the pathogenesis of experimental and natural TSEs.

## Background

Transmissible spongiform encephalopathies (TSEs) are fatal neurodegenerative disorders of the central nervous system in humans and animals [1]. The most widely accepted hypothesis holds that the disease-causing event for naturally acquired TSEs is a peripheral infection with an "infectious" protease-resistant isoform of the prion protein (PrP) that initiates the conversion of the host's cellular PrP into a pathologically misfolded and/or aggregated form, referred to as PrP^Sc ^[2-4] or PrP^TSE ^[5]. The presence of PrP^TSE ^is linked with infectivity and has been established as a reliable biochemical marker for TSE agents in scrapie-infected hamsters as well as in other animal species and humans [6].

The pathophysiology of the spread of TSE agents through the body of peripherally infected individuals has been examined in a variety of host species by monitoring the PrP^TSE ^status in various tissues at different stages of incubation and clinical disease. Depending on the site of peripheral infection, the host species, its genetic PrP background, and the dose and strain of agent, PrP^TSE ^could be detected early in mobile immune cells, *e.g*. dendritic cells [7], in follicular dendritic cells [8] of the lymphoreticular system (LRS) [9,10], in nerves and ganglia of the peripheral nervous system (PNS), and in areas of the central nervous system (CNS) to or from where early infected PNS components project [6,11-14]. Taken together, these findings, and data from further bioassay studies, implicate that lymphoreticular and neural tissues are involved in the centripetal invasion of the CNS upon a peripheral infection with TSE agents.

However, whereas neurally mediated propagation of infection to the brain and spinal cord appears to be a common hallmark of the pathophysiology of peripherally acquired TSEs, findings on the role of the lymphoreticular system in neuroinvasion are less uniform. In sheep carrying a PrP^VRQ/ARR ^genotype, scrapie invasion of the CNS was reported to occur without prior infection of lymphoid tissue [15], and in cattle affected with bovine spongiform encephalopathy (BSE) the distribution of PrP^TSE ^and infectivity in the LRS is strikingly limited [6,16]. On the other hand, functional studies in mice that were infected *via *different peripheral routes with or without splenectomy clearly demonstrated the relevance of the spleen for the centripetal propagation of infection in these murine models [17,18]. Genetically modified mice with a reduced number of Peyer's patches in the small intestine showed a high resistance to infection upon a peroral challenge with scrapie [19], and neuroinvasion of scrapie agent was impaired after skin scarification in lymphotoxin deficient mice lacking lymph nodes draining the skin [20].

In a previous study, we monitored propagation of PrP^TSE ^deposition along the sciatic nerve of Syrian hamsters infected with scrapie *via *the footpad [21]. Here, in the same hamster model of footpad-infection, we examined the status of PrP^TSE ^in the draining lymph node of the foot and performed surgical regional lymphadenectomy. The experiments were carried out in order to elucidate the influence of the draining lymph node on the pathogenesis of scrapie, and to establish whether lethal neuroinvasion can be prevented by post- or pre-exposure lymphadenectomy in our non-murine rodent model of peripheral scrapie infection.

## Methods

### Inoculation and clinical monitoring of animals

The animal experiments complied with German legal regulations and were approved by the responsible authorities. Adult male and female Syrian hamsters were infected under anaesthesia by injection into the right footpad of 20 μl of a 2%, a 1%, a 0.1%, a 0.01% or a 0.001% (w/v) 263 K scrapie hamster brain homogenate from terminally ill donor animals as described elsewhere [21]. 20 μl of the 1% inoculum contained approximately 2–6 × 10^5 ^50% intracerebral lethal doses (LD_50i.c._). Particular caution was taken to avoid injury of blood vessels during the injection of the inoculum. The hamsters were monitored at least twice a week for the development of clinical signs of scrapie. Hamsters diseased with 263K scrapie showed head bobbing, ataxia of gait and generalized tremor. Such animals were frequently and persistently in motion, easily irritated by noise and touch, and had difficulties maintaining balance and rising from a supine position. When terminally affected with scrapie (a disease stage which is accompanied by fully developed clinical symptoms and indications that the animals become unable to take up sufficient quantities of drinking water), at pre-defined time points during the incubation period, or at 314 days after infection at the latest, the hamsters were sacrificed by CO_2 _asphyxiation.

### Lymphadenectomy

For large scale lymphadenectomy, animals were anaesthetized with a Ketamin/Xylazin mixture (100 and 5 mg/kg body weight, respectively). After shaving and skin disinfection in the region of the popliteal fossa of the right hind leg the *Ln. popliteus *was excavated and removed using a thermo-coagulator to avoid bleeding and dissemination of scrapie agent during the surgical intervention. The skin was closed with Vicryl 5.0 suture. For sham-operation, animals were anaesthetised and skin suture was accomplished at the level of the right popliteal fossa.

### Experimental groups and tissue collection

#### PrP^TSE ^detection in regional lymphoid tissue after footpad-infection

After sacrification of animals infected with 20 μl of the 2% inoculum the ipsilateral and contralateral *Ln. popliteus *were removed for detection of PrP^TSE ^by Western blotting (n = 2 for the following time points each: 2 days post infection (dpi), 42-, 70-, 80-, 90- and 100 dpi).

#### Regional lymphadenectomy after footpad-infection

Subsequent to footpad-infection with a high dose or a low dose of 263K scrapie brain homogenate (20 μl of the 1% and 0.01% inoculum, respectively) hamsters were subjected to ipsilateral lymphadenectomy at the following time points after inoculation: 4 h; 24 h; 48 h and 6 dpi (n = 6 for each combination of dose and time point, table 1).

**Table 1:** Survival times of hamsters that underwent regional lymphadenectomy after scrapie infection

	Sham operation 6 days **after **infection		Ectomy 4 h **after **infection		Ectomy 24 h **after **infection		Ectomy 2 days **after **infection		Ectomy 6 days **after **infection	
	individual survival	mean ± SD	individual survival	mean ± SD	individual survival	mean ± SD	individual survival	mean ± SD	individual survival;	mean ± SD

Infectious dose: 20 μl **1%** 263K-homogenate	108; 108; 121; 121; 121; 121	117 ± 7	111; 111; 108; 114; 114; 118	113 ± 3; p ≤ 0.25	104; 108; 108; 111; 114; 114	110 ± 4 p ≤ 0.1	108; 111; 114; 114; 114; 118	113 ± 3 p ≤ 0.5	93; 108; 108; 111; 114; 118	109 ± 9; p ≤ 0.1

Infectious dose: 20 μl **0.01%** 263K-homogenate	108; 111; 115; 118	113 ± 4	100; 108; 111; 115; 118; 115	111 ± 7; p ≤ 1.0	111; 115; 118; 121; 174	128 ± 26; p ≤ 0.5	118; 118; 121; 132; 178	133 ± 26; p ≤ 0.25	115; 115; 125; 125; 128; 128	123 ± 6; p ≤ 0.05*

Individual survival times, i. e. the interval of time between incoculation and the occurrence of terminal disease (days), mean survival times (days ± SD) and p-values (levels of significance as determined vs. the sham-operated control groups) of hamsters that underwent regional lymphadenectomy of the *Ln. popliteus *at the indicated time points following inoculation of a high or a low dose of 263K scrapie agent into the footpad. Sham operations were performed at 6 dpi. Asterisk indicates a statistically significant increase of the mean incubation time as compared to the sham-operated control group (p = 0.05, Student's unpaired two-tailed t-Test).

Note: Individual incubation times until the onset of clinical symptoms (not shown) were approximately 8–14 days shorter than the reported survival times.

Two control groups similarly infected *via *the footpad with 20 μl of the 1% and 0.01% inoculum (n = 6 each) were sham-operated at 6 dpi. Four animals from different groups challenged with the 0.01% inoculum died for reasons unrelated to scrapie or surgical intervention.

#### Regional lymphadenectomy prior to footpad-infection

Four weeks before footpad-infection with a high dose, a medium dose or a very low dose of 263K-scrapie brain homogenate (20 μl of 2%, 0.1% or 0.001% inoculum, respectively) hamsters were subjected to ipsilateral lymphadenectomy. Three control groups similarly infected *via *the footpad with 20 μl of the 2%, 0.1% or 0.001% inoculum were sham-operated four weeks before footpad-infection. The experimental groups consisted of 7–8 animals, each (table 2). One control animal challenged with the 2% inoculum died for reasons unrelated to scrapie or surgical intervention.

**Table 2:** Survival times of hamsters that underwent regional lymphadenectomy before scrapie infection

	Sham operation 4 weeks **before **infection		Ectomy 4 weeks **before **infection	
	individual survival	mean ± SD	individual survival	mean ± SD

Infectious dose: 20 μl **2%** 263K-homogenate	105; 105; 108; 115; 115; 122	112 ± 7	108; 112; 112; 115; 115; 115	114 ± 4

Infectious dose: 20 μl **0.1%** 263K-homogenate	131; 138; 168; 176 176; 182; 182; 200	169 ± 23	125; 127; 127; 131; 134; 168; 182; 218	155 ± 34

Infectious dose: 20 μl **0.001%** 263K-homogenate	219; 247; 247; 288; 300; 308; 314*	268 ± 36 (without *)	308; 314*; 314*; 314*; 314*; 314*; 314*	

Individual survival times (days) and mean survival times (days ± SD) of hamsters that underwent regional lymphadenectomy of the *Ln. popliteus*, or sham-operation, four weeks before footpad infection with a high, a low or a very low dose of 263K-scrapie agent. Asterisk indicates animals that did not show onset of scrapie symptoms until the termination of the experiment at 314 dpi.

### Extraction of PrP^TSE ^and Western blotting

Tissue extraction of PrP^TSE ^in the form of protease-resistant PrP27–30 was started by collagenase digestion of samples for one hour and subsequently performed as previously published [22]. The extracts were subjected to polyacrylamide gelelectrophoresis and Western blotting using the anti-PrP monoclonal antibody 3F4 [23] as described in detail elsewhere [22,24].

### Paraffin-embedded tissue (PET) blotting

**Figure 1:**
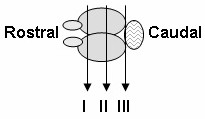
Schematic representation of a hamster brain, top view. Levels of coronal brain slices (I, II and III) used for PET blot analysis of cerebral PrP^TSE ^deposition in animals that were sham-operated or lymphadenectomized four weeks before footpad-infection with a 0.001% 263K scrapie brain homogenate.

PET blot analysis of PrP^TSE ^deposition in coronal brain slices representing four different cerebral locations (levels I, II and III according to figure 1, and one region containing the red nucleus from which neural projections extend to the sciatic nerve innervating the footpad) of hamsters that were sham-operated and lymphadenectomized four weeks before footpad infection with the 0.001% inoculum was performed as described by Thomzig *et al*. [25].

## Results

### Western blot detection of PrP^TSE ^in the regional lymph node after footpad- infection

**Figure 2:**
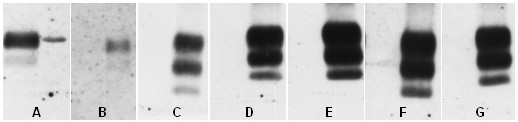
Western blot detection of PrP27–30, the Proteinase K-resistant core of PrP^TSE^, in lymphonodal tissue (*Ln. popliteus) *at different time points after infection of hamsters *via *the footpad with a 2% 263K-scrapie brain homogenate. The amount of tissue subjected to testing is specified in brackets. **A**, left lane: blot control, 263K-scrapie hamster brain homogenate containing 10^-6 ^g of brain tissue; right lane: 27kD marker; **B**, 2 dpi, left lane: contralateral *Ln*. (3.1 mg); right lane: ipsilateral *Ln*. (3.3 mg); **C**, 42 dpi, left lane: contralateral *Ln*. (2.9 mg); right lane: ipsilateral *Ln*.(4.8 mg); **D**, 70 dpi, left lane: contralateral *Ln*. (7.0 mg); right lane: ipsilateral *Ln*. (7.4 mg); **E**, 80 dpi, left lane: contralateral *Ln*. (7.9 mg); right lane: ipsilateral *Ln*. (4.7 mg); **F**, 90 dpi, left lane: contralateral *Ln*. (4.1 mg); right lane: ipsilateral *Ln*. (4.3 mg); **G**, 100 dpi, left lane: contralateral *Ln*.(1.9 mg); right lane: ipsilateral *Ln*.(3.9 mg).

A time course study by Western blot analysis of PrP^TSE ^deposition in the ipsilateral draining *Ln. popliteus *showed early and ongoing accumulation of the pathological prion protein after footpad-infection with the 2% scrapie inoculum. Figure 2 shows representative findings from animals at 2- (2), 42- (1), 70- (2), 80- (2), 90- (2) and 100 dpi (2) (numbers in brackets: positive results found with 2 animals examined at each time point). However, in the contralateral *Ln. popliteus*, PrP^TSE ^could not be detected at any time point (figure 2).

### Effect of regional lymphadenectomy performed after footpad-infection

The minimal-invasive surgical intervention was well tolerated by all animals. Regional lymphadenectomy of the draining *Ln. popliteus *at different time points after footpad-infection with a high dose of scrapie agent (1% inoculum) produced no statistically significant difference in the mean survival time (*i. e*. the interval of time between infection and the occurrence of terminal scrapie symptoms) as compared to the sham-operated control group (table 1). However, as can be seen from table 1, the situation looks different after footpad-infection with the low-dose (0.01%) inoculum. Here, one conspicuous outlier each occurred when lymphadenectomy was performed at 24 h and 2 days after infection, with highly prolongated survival times of 174 and 178 days, respectively. Furthermore, when lymphadenectomy was performed at 6 days after the low-dose challenge, a statistically significant increase in the mean survival time as compared to the control group was found.

### Effect of regional lymphadenectomy performed four weeks before footpad-infection

**Figure 3:**
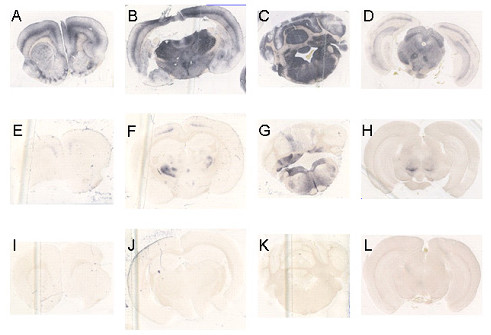
PET blot analysis of PrP^TSE ^deposition in coronal brain slices of hamsters that were sham-operated or lymphadenectomized four weeks before footpad infection with the 0.001% inoculum. Levels correspond to regions I-III as indicated in figure 1 (A-C, E-G, I-K), and to a region containing the red nucleus (from where neural projections extend to the sciatic nerve innervating the footpad [D, H, L]). A-D: Samples from a sham-operated hamster that developed terminal scrapie at 300 dpi. E-H: Samples from the sham-operated hamster that remained free of scrapie symptoms until 314 dpi. I-L: Samples from one animal representative for five lymphadenectomized hamsters that remained free of scrapie symptoms until 314 dpi.

As compared to the respective control groups, survival times remained virtually identical or did not show a statistically relevant difference when the ipsilateral *Ln. popliteus *was removed four weeks prior to footpad-infection with a high (2% inoculum) or medium dose (0.1% inoculum) of infectivity, respectively (table 2). However, a pronounced effect could be observed when the footpad-infection was performed after lymphadenectomy with a very low dose of agent, *i. e*. the 0.001% inoculum. Here 6 out of 7 sham-operated control animals showed beginning or terminal scrapie after 258 ± 39 days (incubation time) and 268 ± 36 dpi (survival time [mean ± SD], table 2), respectively. Only one animal of this control group remained free of scrapie symptoms until termination of the experiment at 314 dpi. In contrast, 6 out of 7 lymphadenectomized hamsters stayed free of clinical scrapie until 314 dpi. A statistical analysis using a contingency table (table 3) and Fisher's exact test (Graphpad-Prism-Software) revealed a low probability of only 2.91% that the observed discrepancy between the lymphadenectomized and control group was statistically irrelevant. This formally confirms that sham-operated hamsters are at much higher risk to develop scrapie during the period of observation than their lymphadenectomized counterparts. Consistent with the results of the statistical analysis, PET blot examination of the brains from symptom-free lymphadenectomized hamsters sacrificed at 314 dpi showed absence of detectable cerebral PrP^TSE ^deposition in five out of six animals (figure 3, I–L). In contrast, all animals of the sham-operated group – including the hamster that did not show scrapie symptoms until 314 dpi – displayed pronounced cerebral PrP^TSE ^deposition in the PET blots (figure 3, A–H).

**Table 3:** Contingency table

Intervention (four weeks **before **infection)	Development of clinical disease until 314 dpi		Number of animals tested
	Yes	No	
Sham operation	**6**	**1**	**7**
Lymphadenectomy	**1**	**6**	**7**

Table of contingency indicating the development of clinical disease in animals which underwent ipsilateral lymphadenectomy or sham operation four weeks before footpad infection with 0.001% scrapie-inoculum.

## Discussion

Following a peripheral infection with TSE agents, initial PrP^TSE ^deposition and replication of infectivity within the host was found to occur in components of the LRS and PNS [6,9,26-29]. In the light of these findings, a two-phase model has been suggested for prion neuroinvasion after peripheral uptake of agent. This model postulates the following sequence of events [30,31]: 1^st^) replication of infectivity in the LRS, and 2^nd^) neuroinvasion by transfer of prions to components of the LRS innervation. However, other findings suggest that direct infection of the nervous system is possible independently of the LRS, especially when the infection is caused by a high doses of agent (for review see: [6]). Against this background, the intention of our study was to further pinpoint the relevance of draining lymphoid tissue for the spread of prions from a peripheral site of inoculation to the CNS in a qualified *in vivo *model using high and low doses of scrapie agent for infection prior to, and after lymphadenectomy.

When we performed a unilateral footpad infection of hamsters with scrapie in a previous study, PrP^TSE ^could be detected in the ipsilateral sciatic nerve at about 60 dpi, before the onset of clinical symptoms. In the contralateral sciatic nerve, however, detection of PrP^TSE ^was possible only at about 80 dpi or later [21]. This pointed to an ipsilaterally centripetal and subsequent contralaterally centrifugal sciatic propagation of the infection. Here, we extended our studies to the regional lymphoid tissue and detected an accumulation of PrP^TSE ^in the footpad-draining *Ln. popliteus *from the first time point of investigation, *i. e*. 2 dpi, onwards (right lane in B of figure 2). In contrast, the corresponding contralateral lymph node was consistently negative for PrP^TSE ^at all examined time-points (figure 2), showing that a systemic lymphonodal generalisation of infection did not appear until the terminal stage of the disease. In this context it should also be noted that Western blot detection of PrP^TSE ^in the spleens of our animals was not possible before 60 dpi (data not shown).

Bearing in mind the draining function of regional lymph nodes at sites of peripheral infection, early accumulation of PrP^TSE ^in the ipsilateral *Ln. popliteus *of our footpad-infected model animals is not surprising. Moreover, accumulation of PrP^TSE ^in the regional lymphonodal system was demonstrated previously in different *in vivo *models, *e.g*. in mice unilaterally exposed to scrapie agent *via *the skin [32]. Also, hamsters perorally challenged with scrapie accumulated PrP^TSE ^within the first days after inoculation in Peyer's patches [33].

Despite the consistent detection of PrP^TSE ^in the regional *Ln. popliteus *of our model animals at 2 dpi and all following time points, resection of this lymph node performed at 4 h, 24 h, 2- or 6 days after administration of the high dose of agent yielded no differences in the observed survival times or attack rates as compared to the control groups. However, similar intervention at 24 h and 2 days after low-dose infection produced substantially increased incubation times in one out of 5 animals per group, each (table 1). Such effect was never observed when animals were inoculated with the high dose of agent and indicates that the *Ln. popliteus *may influence pathogenetic events following a low-dose challenge at least in a subset of animals. Furthermore, when lymphadenectomy was performed at 6 days after low dose infection, a statistically significant increase of the mean survival time of 10 days was observed. The various effects of lymphadenectomy performed at different time points may have resulted from a possible reduction of the load of infectivity below a critical threshold or a variably efficient "transition-block" before the agent had accomplished neuroinvasion. However, the interpretation of the data obtained when regional lymphadenectomy had been performed after footpad infection may be complicated by interfering effects of the surgical intervention itself. Conspicuously, the sham-operated animals that were inoculated with the 0.01% scrapie brain homogenate did show virtually the same survival time as the sham-oparated hamsters challenged with 100 times more infectivity (table 1). Typically, the incubation period or survival time is indirectly proportional to the administered dose of agent. The absence of this well-established dose-response relation in our experiment may have resulted from inflammatory conditions that were present for a certain time at the site of operation. Such inflammatory or other pathological processes may have considerably accelerated the progress of infection in the low-dose group, whereas the high-dose challenge left less space for a further reduction of the incubation or survival time by additional factors.

In order to avoid interfering effects from surgery, and in order to broaden the range of infectious doses used for footpad infections we resorted to a modified experimental paradigm. In these follow-up experiments we investigated the effect of regional lymphadenectomy performed four weeks before footpad-infection with a 2%-, a 0.1%- and a 0.001% inoculum. In these animals inflammatory or other pathological processes due to incomplete healing of the operation wound should be absent, and, indeed, here the survival times of the hamsters showed a normal inverse relation to the three administered doses of agent (table 2). The accelerating effect of surgical intervention around the time point of infection as discussed above is highlighted by the fact that hamsters which were sham-operated at 6 days after footpad-infection with an 0.01% inoculum showed a considerably shorter survival time than hamsters which received a ten times higher dose of agent (0.1% inoculum) four weeks after the sham-operation (113 ± 4, table 1, *vs*. 169 ± 23 days, table 2). In addition, with the 0.001% inoculum, a pronounced delaying effect on the propagation of infection was observed when lymphadenectomy had been performed four weeks prior to footpad-infection. While six out of seven sham-operated controls showed onset of scrapie at 258 ± 39 dpi, and one symptom-free animal of this group displayed distinct PrP^TSE ^deposition in the brain at 314 dpi (figure 3, E–H), six out of seven lymphadenectomized hamsters remained free of clinical symptoms until 314 dpi (when the experiment was terminated), and the brains of five of these animals were free of detectable PrP^TSE ^as revealed by PET blotting (see representative example in figure 3, I–L).

## Conclusion

Taken together, our interventional study did not show any discernible pathogenetic influence of the regional lymph node on preventing or mediating neuroinvasion of scrapie agent when a relatively high dose (2%, 1%, or 0.1% inoculum) of scrapie agent was administered prior to, or after lymphadenectomy. Indeed, recent studies using the same hamster model and similar doses of agent revealed the regional nerve (*N. ischiadicus*) as the prime pathway for CNS invasion of the scrapie agent after footpad-infection, as evidenced by a substantial prolongation of survival after neurectomy (Kratzel *et al*., submitted for publication).

However, findings from our first experimental series reported here indicated that the *Ln. popliteus *may have modulated the incubation time in a limited subset of animals upon low dose infection with the 0.01% inoculum when lymphadenectomy was performed at different time points after footpad-infection. In these experiments, our findings suggested the regional lymph node as a potentially facilitating factor contributing to neuroinvasion. This conclusion was confirmed and expanded by the results of our second set of experiments, when a very low dose of agent (0.001% inoculum) was administered to hamsters that were lymphadenectomized four weeks before infection: Under these conditions lymphadenectomy prevented detectable cerebral prion invasion – as monitored by PET blotting – in a large proportion of animals (table 2 and figure 3).

It has been well established that the lymphoid pathogenesis of acquired TSEs depends on a variety of parameters such as the host species, the strain and dose of agent, or the route of infection [6]. Contrary effects of splenectomy have been observed in mice and hamsters [18,34], and Glaysher and Mabbott [20] reported impaired neuroinvasion after scrapie infection *via *the skin of genetically modified mice lacking draining lymph nodes. We suggest the application of our invasive *in vivo *model in mice and other animal species to further elucidate the involvement of lymphoid tissue in the pathogenesis of experimental and natural TSEs.

## Competing interests

The author(s) declare that they have no competing interests.

## Authors' contributions

CK, DK and MB conceived and designed the experiments. CK and DK performed the experiments. CK, DK and MB analyzed the data and wrote the paper.
